# Exploring the Limits of Combined Image/'omics Analysis for Non-cancer Histological Phenotypes

**DOI:** 10.3389/fgene.2020.555886

**Published:** 2020-10-23

**Authors:** Paul Gallins, Ehsan Saghapour, Yi-Hui Zhou

**Affiliations:** ^1^Bioinformatics Research Center, North Carolina State University, Raleigh, NC, United States; ^2^Department of Biological Sciences, North Carolina State University, Raleigh, NC, United States

**Keywords:** imaging, genomics, pathology, prediction, integration, histology, machine learning, embedding

## Abstract

The last several years have witnessed an explosion of methods and applications for combining image data with 'omics data, and for prediction of clinical phenotypes. Much of this research has focused on cancer histology, for which genetic perturbations are large, and the signal to noise ratio is high. Related research on chronic, complex diseases is limited by tissue sample availability, lower genomic signal strength, and the less extreme and tissue-specific nature of intermediate histological phenotypes. Data from the GTEx Consortium provides a unique opportunity to investigate the connections among phenotypic histological variation, imaging data, and 'omics profiling, from multiple tissue-specific phenotypes at the sub-clinical level. Investigating histological designations in multiple tissues, we survey the evidence for genomic association and prediction of histology, and use the results to test the limits of prediction accuracy using machine learning methods applied to the imaging data, genomics data, and their combination. We find that expression data has similar or superior accuracy for pathology prediction as our use of imaging data, despite the fact that pathological determination is made from the images themselves. A variety of machine learning methods have similar performance, while network embedding methods offer at best limited improvements. These observations hold across a range of tissues and predictor types. The results are supportive of the use of genomic measurements for prediction, and in using the same target tissue in which pathological phenotyping has been performed. Although this last finding is sensible, to our knowledge our study is the first to demonstrate this fact empirically. Even while prediction accuracy remains a challenge, the results show clear evidence of pathway and tissue-specific biology.

## 1. Introduction

Histopathology refers to the microscopic examination of tissues in order to identify possible changes caused by disease, which is still largely conducted by human pathologists using expert judgment. High resolution imaging has made it possible to use machine learning to perform histopathological assignment. Moreover, outside of cancer diagnostics, few studies have attempted to combine histological phenotypes with genomic measurements due to the lack of available datasets. The Genotype-Tissue Expression (GTEx) project includes histology image data, with expert pathological classification, and RNA-seq expression data. The Biorepositories and Biospecimen Research Branch (BBRB) of the Cancer Diagnosis Program (CDP) at the National Institute of Health (NIH) manages the collection of tissue and blood biospecimens for GTEx from over 900 deceased donors who were identified through organ and tissue transplant programs (https://biospecimens.cancer.gov/resources/sops/gtex.asp). The BBRB has adopted standard operating procedures for various biobanking practices. One is a protocol for the uniform histologic analysis of GTEx tissue specimens and for generating a case summary report for the tissues received and evaluated. The Comprehensive Biospecimen Resource receives and processes these tissue specimens and generates digital images. A certified pathologist reviews the tissue images to confirm the presence of any pathologic findings.

The design of GTEx makes it an ideal testing ground for the difficult problem of imaging- and expression-based prediction of subclinical pathologies. The use of machine learning for image and genomic analysis in cancer tissue diagnostics is well-established (Mobadersany et al., 2018; Halama, 2019), but has not been well-developed for subclinical pathologies, for which pathological specimens are unlikely to be available. Two reports have performed limited imaging/expression analysis within one or two GTEx tissues at a time, for a corresponding pathological designation. Barry et al. (2018) examined 341 samples in thyroid, and focused primarily on image feature extraction to predict a pathological designation, and Ash et al. (2018) examined colon and thyroid similarly, where the focus was primarily on genomic associations with extracted image components rather than pathology directly. The motivation for GTEx (Lonsdale et al., 2013) was that the expression quantitative trait locus dissection of disease pathology is best performed using expression in the same tissue manifesting the pathology. The expression QTL results from GTEx v8 (GTEx Consortium et al., 2020) provide incomplete support for this hypothesis, as a large proportion of significant eQTLs appear to be common across tissues, raising the possibility of analogous findings for histopathological designations. In other words, it is unclear whether expression should be measured in the *same* tissue as that providing the basis for diagnosis.

In addition to uncertainty described above, previous work has left unanswered the question of whether genomic measurements, images, or a combination of the two provide the best predictive ability for a sub-clinical pathology. Genomic measurements provide greater biological interpretability than imaging, and so might be preferred in many circumstances if tissues are available.

Here we perform a comprehensive investigation of six pathological designations in five GTEx tissues, exploring the limits of machine-learning prediction accuracy using imaging data, expression, and their combination.

## 2. Data Preparation

### 2.1. Histopathological Data

Original GTEx histology images were downloaded from the Biospecimen Research Database (https://brd.nci.nih.gov/brd/image-search/searchhome). These image files are in Aperio SVS format, a single-file pyramidal tiled TIFF. The RBioFormats R package (https://github.com/aoles/RBioFormats), which interfaces the OME Bio-Formats Java library (https://www.openmicroscopy.org/bio-formats), was used to convert the files to JPEG format, and these images were processed using the Bioconductor package EBImage (Pau et al., 2010). Following the method proposed by Barry et al. (2018) to segment individual tissue pieces, the average intensity across color channels was calculated, and adaptive thresholding was performed to distinguish tissue from background. A total of 117 Haralick image features were extracted from each tissue piece by calculating 13 base Haralick features for each of the three RGB color channels and across three Haralick scales by sampling every 1, 10, or 100 pixels. After removing overly small tissue pieces, feature values were averaged across pieces for each sample. Then features were log2-transformed and normalized to ensure feature comparability across samples. Pathology data for all histology samples are available on the GTEx Portal (https://www.gtexportal.org/home/histologyPage). Sex and age are also provided. Six pathology categories in five tissues were selected, based on completeness of data: fibrosis in lung (*n* = 831 for image, *n* = 513 for expression), congestion in liver (*n* = 600, *n* = 205), steatosis in liver (*n* = 600, *n* = 205), atherosclerosis/atherosis/sclerotic in tibial artery (*n* = 836, *n* = 508), Hashimoto's thyroiditis in thyroid (*n* = 892, *n* = 570), and fibrosis in adipose tissue (*n* = 963, *n* = 574). Each phenotype was coded as presence (coded 1) or absence (0) of a particular pathology.

### 2.2. Gene Expression Data

For each tissue type, a subset of subjects also had gene expression data from RNA-Seq. The v8 release is available on the GTEx Portal (https://www.gtexportal.org/home/datasets). Gene read counts were normalized between samples using TMM, and genes were selected based on expression thresholds explained in GTEx Consortium et al. (2020). To account for hidden batch effects in the gene expression data, GTEx implemented the Probabilistic Estimation of Expression Residuals (PEER) method (Stegle et al., 2010) to estimate a set of cofactors for each tissue type. This approach builds on factor analysis methods that infer broad variance components in the measurements. It outputs hidden cofactors that explain much of the expression variability among individuals. These PEER cofactors are treated as covariates in association models to increase power for detecting expression traits. Using a standard approach for our expression analyses, PEER cofactors that were significantly associated with a phenotype (false discovery *q* < 0.1) were included, along with sex, as covariates.

## 3. Analyses

### 3.1. Integrative Analyses

In order to best represent prediction accuracy for relatively interpretable models, we used a combination of principal components and LASSO regression as initial analyses with cross-validation, and area under the receiver-operator characteristic curve (AUC) as the performance criterion. To explore the limits of prediction accuracy, we also performed a suite of additional machine learning prediction approaches.

#### 3.1.1. Initial Prediction

To reduce the number of image features, principal components analysis (PCA) was performed as described (Barry et al., 2018), and the first 10 PCs were included in downstream analyses. Logistic regression was performed to test if the image PCs alone can predict pathology by the following methods: (1) including all observations in a model and output the predicted values directly; and (2) 50 iterations of five-fold cross-validation and output the average predicted values. We computed the AUCs between the predicted values and the phenotype. In a similar manner as Barry et al. (2018), we subsampled the original images down to 1,000 × 1,000 pixels to illustrate the predictive performance of the ten image PCs in a logistic regression model. Then we tested if gene expression alone can predict pathology by running fifty iterations of five-fold cross-validation of LASSO logistic regression. Within each fold, we ran the cv.glmnet function in R (alpha=1) on the training set and selected the gene predictors which gave non-zero coefficients at the lambda with the smallest mean cross-validated error. Finally, we ran an integrative analysis of image features and gene expression to predict pathology. Here the strategy was to combine the ten imaging PCs with the gene-expressed predictors selected by LASSO into a single generalized linear regression model, again using fifty iterations of five-fold cross validation. For each model, analyses were performed both without and with covariates. For imaging prediction, the covariates were age and sex, and additional covariates for expression prediction were the PEER cofactors as described.

#### 3.1.2. A Larger Suite of Prediction Approaches

**Figure 1:**
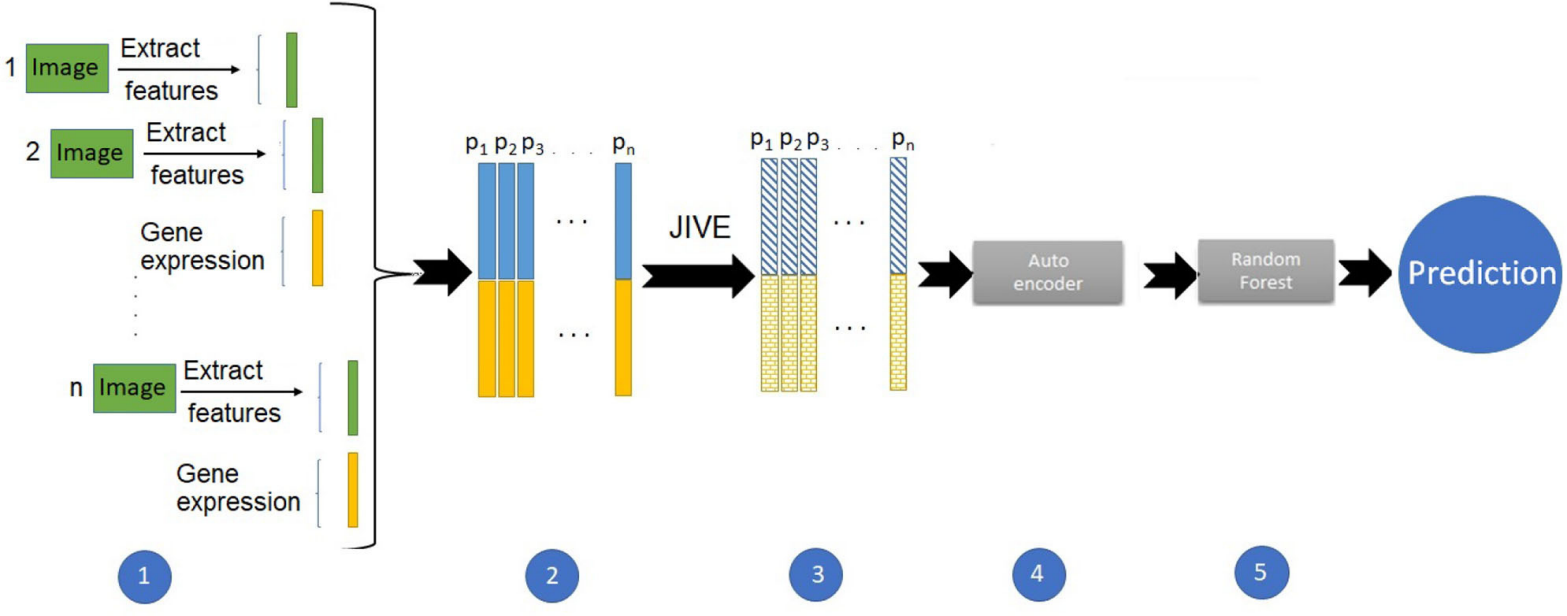
Pipeline to build a prediction model with integrated imaging and expression data.

We started with the same set of 10 PCs from the image features. We also ran PCA on the gene expression data, and due to the higher dimensionality, we included the first twenty expression PCs in the predictive models. For the predictions using image and expression separately, we ran a suite of six supervised machine learning methods: random forest (RF), support vector machines (SVM), naive Bayes (NB), linear discriminant (LD), quadratic discriminant (QD), and logistic regression (LR). We used the Classification Learner application in Matlab to train these models with five-fold cross-validation and for classification. Figure 1 illustrates the pipeline developed for the integrative analysis of image and gene expression features. Combining these data types as input, we applied the joint and individual variation explained (JIVE) method (Lock et al., 2013), which partitions joint and individual sources of variation between data types. Our approach used an autoencoder, a type of artificial neural network, to reduce the dimensionality of the data. We chose the first twenty features from this method as input to build a predictive model using random forests.

#### 3.1.3. Pathway Analysis

We ran a regression analysis of pathology phenotype against gene expression in each tissue, and selected the subset of significant genes (false discovery *q* < 0.1) as input for pathway analysis. We ran the Ingenuity Pathway Analysis (IPA) software (QIAGEN Inc., https://digitalinsights.qiagen.com/products-overview/discovery-insights-portfolio/analysis-and-visualization/qiagen-ipa) and the DAVID functional annotation tool (Huang et al., 2009a,b). Both methods test if pathways, annotated by gene sets in their respective databases, are enriched for significant phenotype-associated genes. Both approaches uses Fisher's exact *p*-values for enrichment of pathway membership compared to the significant gene set. For IPA, we used human genes in the Ingenuity Knowledge Base as the background gene set, along with its default analysis settings. For DAVID, we used all human genes in their database as the background gene set, also with its default settings. Pathways from IPA and functional gene groups from DAVID with FDR *q* < 0.1 were declared significantly enriched for significant genes.

#### 3.1.4. Network Embedding Analysis

**Figure 2:**
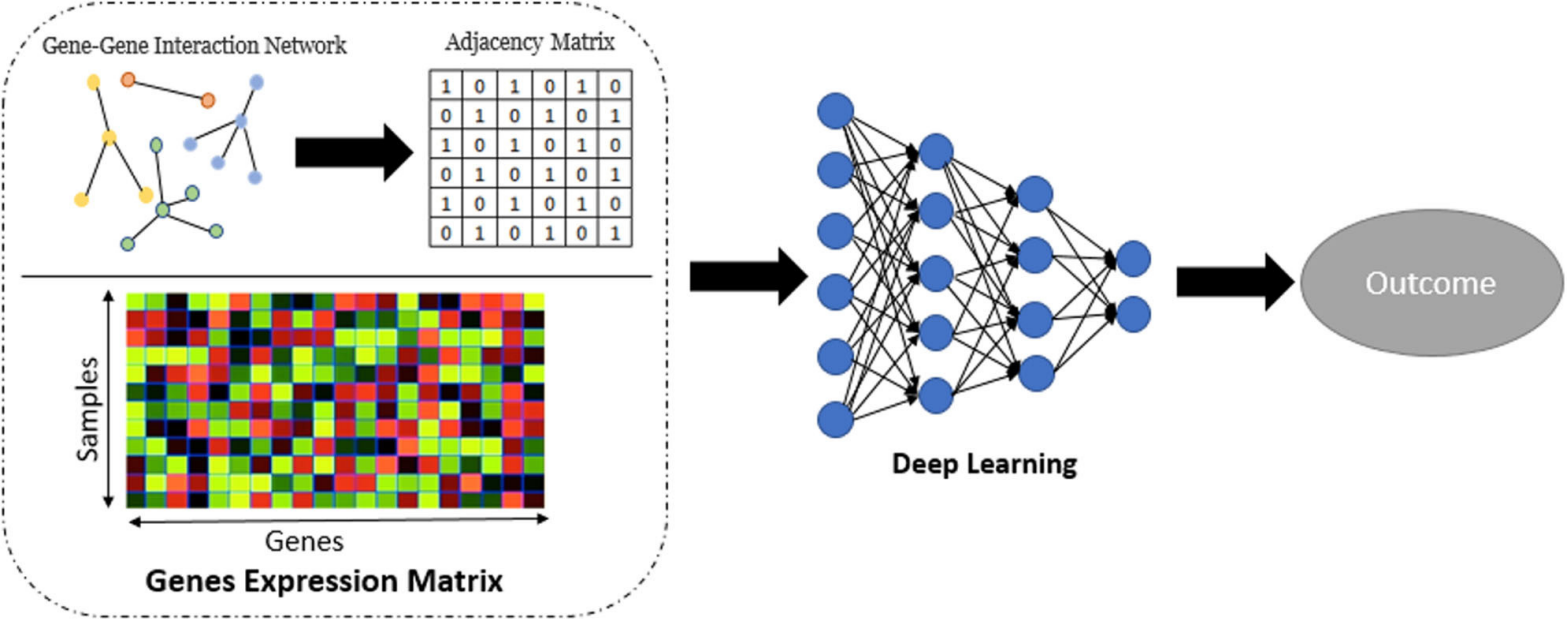
Workflow for the network embedding of gene expression.

Network embedding (Nelson et al., 2019) has numerous applications in computational biology. Although the use of embedding in biological network analysis offers potentially simplified interpretation, we employ it here primarily for its potential robustness/denoising properties (Wang et al., 2018), which may potentially improve prediction. In this part of work, we applied Graph-Embedded Deep Feedforward Networks (GEDFN) (Kong and Yu, 2018) which integrate (embed) an external gene network into the deep neural network architecture. The GEDFN model has a structure of regular neural network architecture with the difference that it applies a gene network called graph-embedding layer instead of the fully connected layer between the input layer and the first hidden layer. In the GEDFN model, the resulting network is represented by a square adjacency matrix, a feature graph which indicates whether pairs of genes are adjacent or not in the network. it comes from the HINT database (http://hint.yulab.org) which is a collection of high-quality protein interactomes from several interactome resources (Das and Yu, 2012). Figure 2 illustrates network embedding workflow. In our approach, since there are the large number of predictors (genes), we considered only the genes which have *p*-value < 0.05 to build the gene network. Supplementary Table 1 shows the size of the gene network for all tissues based on the types of diseases. Then, the input layer of these Deep Feedforward Networks is the gene expression matrix which feeds into a graph-embedded layer. The second and third (hidden) layers are the standard 64-dimensional and 16-dimensional, respectively, and the output layer is 2-dimensional. The Rectified Linear Unit (ReLU) (Nair and Hinton, 2010) was used as the activation function for the model. The Adam optimizer (Kingma and Ba, 2014), an extension of classical stochastic gradient descent, was selected to update network weights iteratively in the training data. Also, five-fold cross validation was used to avoid overfitting.

### 3.2. Cross-Tissue Pathology vs. Expression

For cross-tissue comparisons, we selected the 30 GTEx tissues with both gene expression and imaging data and compared gene expression relationships to the six pathologies and their image features. For each gene, we ran four regression models: phenotype and each of the first three image PCs against expression, including sex as a covariate. The number of samples varied widely by tissue, creating potential differences in power to detect association. Thus, we used the *q*-value package in R to estimate the proportion of non-null *p*-values (π_1_ = 1 − π_0_) in each model, using the overall *p*-value for the collection of imaging PCs. The value π_1_ was used as an estimate of overall expression vs. phenotype relationship (Storey, 2003) that should be relatively insensitive to the sample size.

## 4. Results

**Figure 3:**
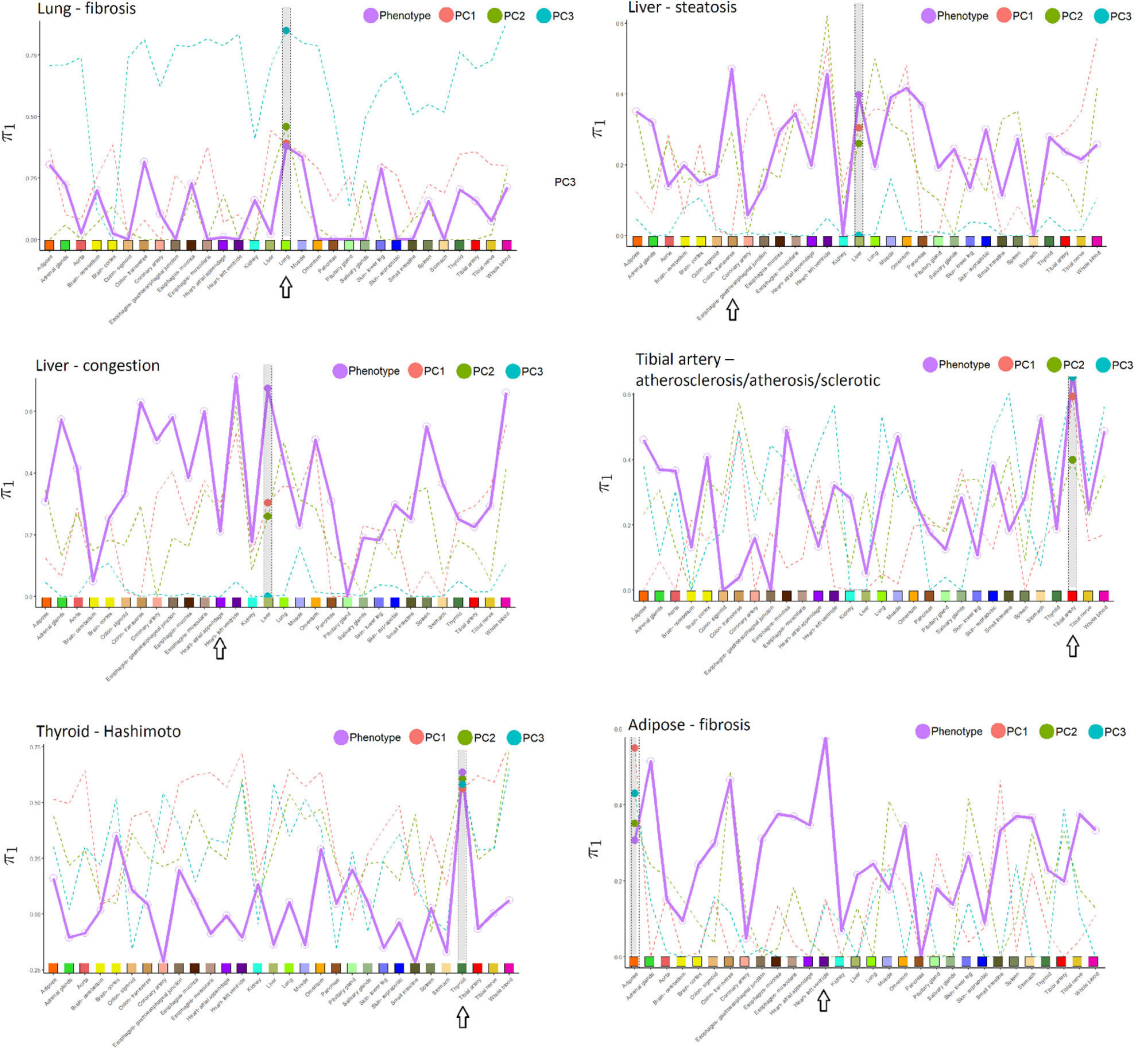
Proportion of non-null *p*-values (π_1_) in the cross-tissue regression analysis of pathology phenotype vs. gene expression, and for image PCs 1–3 vs. expression. Larger dots are the π_1_ values corresponding to the same tissue in which pathology was determined, which is also highlighted in gray in each subfigure. Arrows indicate the tissue with the highest π_1_ for phenotype, in many instances coinciding with the phenotype tissue. Tissues used in the cross-tissue analysis are labeled using the same color scheme used by GTEx Consortium et al. (2020).

We first highlight findings from individual tissues/pathologies, and then provide an overall summary. Table 1 provides summaries of predictive performance for histopathology-derived phenotypes, gene expression data, and integrative analyses. Figure 3 shows the π_1_ values each tissue/pathology vs. the tissue in which expression is measured. Of the six pathologies, only lung fibrosis, liver congestion, tibial artery atherosclerosis, and thyroid Hashimoto's disease resulted in any pathways significant at false discovery *q* < 0.01.

**Table 1:** Summaries of predictive performance for histopathology-derived phenotypes from imaging data, gene expression data, and integrative analyses.

	**Image**				**Expression**					**Combined**	
		**Phenotype**	**Initial**	**Suite**		**Phenotype**	**Initial**	**Suite**	**Embeeding**	**Initial**	**Suite**
**Tissue—pathology**	***N***	**Yes/No**	**AUC**	**AUC**	***N***	**Yes/No**	**AUC**	**AUC**	**AUC**	**AUC**	**AUC**
Lung—fibrosis	831	140/691	0.61	0.62 (RF)	513	74/439	0.63	0.65 (RF)	0.68	0.62	0.57
Liver—steatosis	600	260/340	0.73	0.73 (QD)	205	96/109	0.75	0.74 (RF)	0.81	0.75	0.71
Liver—congestion	600	259/341	0.70	0.69 (NB)	205	80/125	0.76	0.76 (SVM)	0.79	0.76	0.69
Tibial artery—atherosclerosis/atherosis/sclerotic	836	216/620	0.76	0.76 (RF)	508	113/395	0.77	0.76 (LD)	0.76	0.77	0.69
Thyroid—Hashimoto	892	71/821	0.89	0.87 (SVM)	570	37/533	0.95	0.96 (SVM)	0.93	0.96	0.82
Adipose—fibrosis	936	137/826	0.57	0.58 (RF)	574	73/501	0.78	0.68 (LR)	0.84	0.77	0.58

In Table 1, columns 2–3 represent the number of individuals *n* with imaging data for a given tissue, along with case/control counts for the corresponding pathology. Column 4 shows the performance as measured by the area under the receiver-operator characteristic (ROC) curve (AUC) from the regression model of pathology against the 10 image PCs. All of the ROC curves are shown in Supplementary Figure 1. As the ROC curves do not show many instances of crossing, except for curves that are similar throughout, so the AUC is a reasonable summary of performance. Column 5 of Table 1 is the best AUC from among the suite of machine learning methods. Columns 6–7 represent the subset of individuals that also have gene expression data. Column 8 is the AUC from the LASSO regression model of pathology against the significant gene predictors. Column 9 is the best AUC from among the suite of machine learning methods using 20 expression PCs to predict pathology. Column 10 is the AUC from the network embedding analysis with gene expression networks. Column 11 is the AUC from the integrative regression model of pathology against the 10 image PCs and the significant gene predictors. Column 12 is the AUC from the integrative JIVE method. The AUCs for each method without covariates are shown, as they were similar to the covariate-corrected values.

### 4.1. Lung—Fibrosis

Relative to other tissues, the AUCs for lung fibrosis were relatively low. One explanation could be that these images appear to have less definition, resulting in less informative texture features and possibly more difficult to determine the pathology. Nonetheless, in Figure 3, π_1_ for the association of fibrosis against gene expression across tissues was largest for the lung. Using IPA pathway analysis for lung fibrosis, two pathways were significant at *q* < 0,01, *Phagosome Maturation* and *Autophagy* (Supplementary File 1).

### 4.2. Liver—Steatosis and Congestion

The AUCs were modest for both the steatosis and congestion pathologies. For steatosis, the AUC increased substantially using network embedding compared to the other analyses involving expression or image alone. For congestion, predictions using expression alone were markedly higher than using images alone. Using DAVID pathway analysis for liver congestion, two pathway clusters were significant, including those related to intracellular organelle and nuclear lumen and the Golgi apparatus (Supplementary File 2).

### 4.3. Tibial Artery - Atherosclerosis

**Figure 4:**
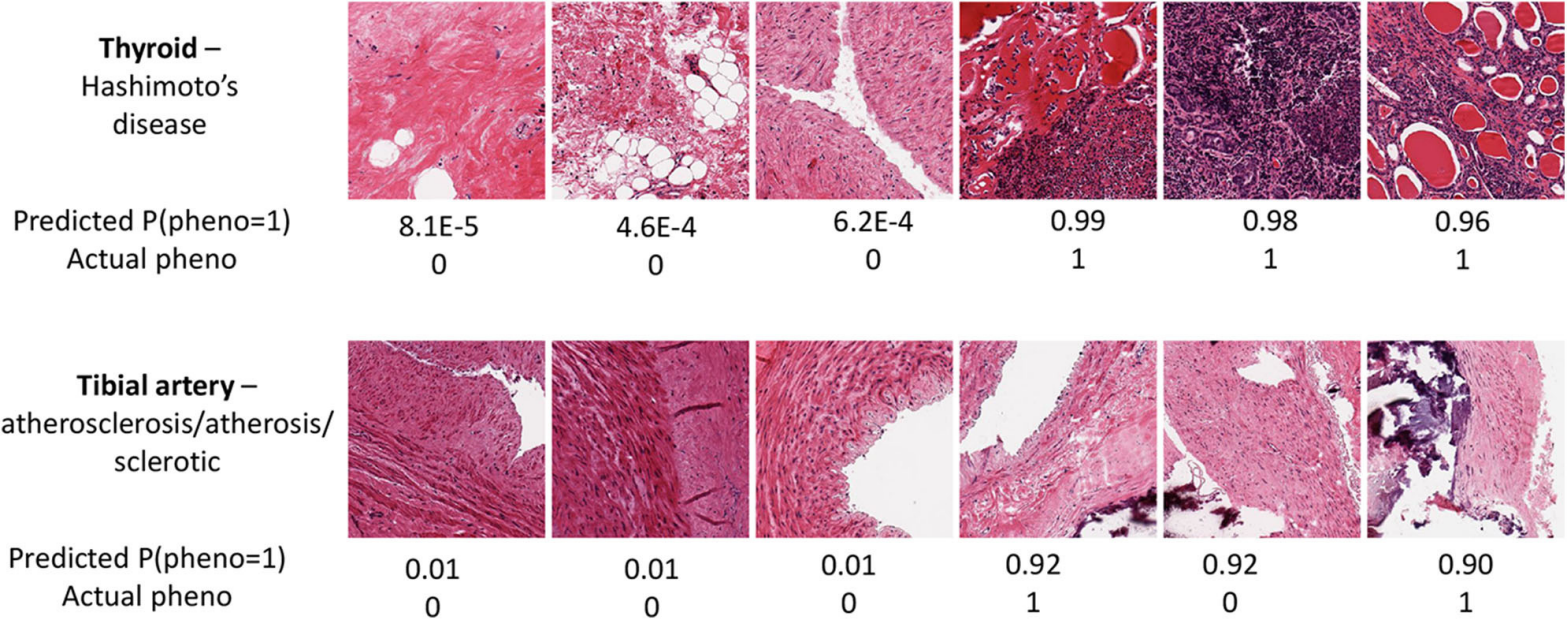
Image samples for two tissues and associated pathologies, for the samples with the three highest and lowest predicted probabilities for thyroid Hashimoto's disease **(Top)** and tibial artery atherosclerosis **(Bottom)**.

**Figure 5:**
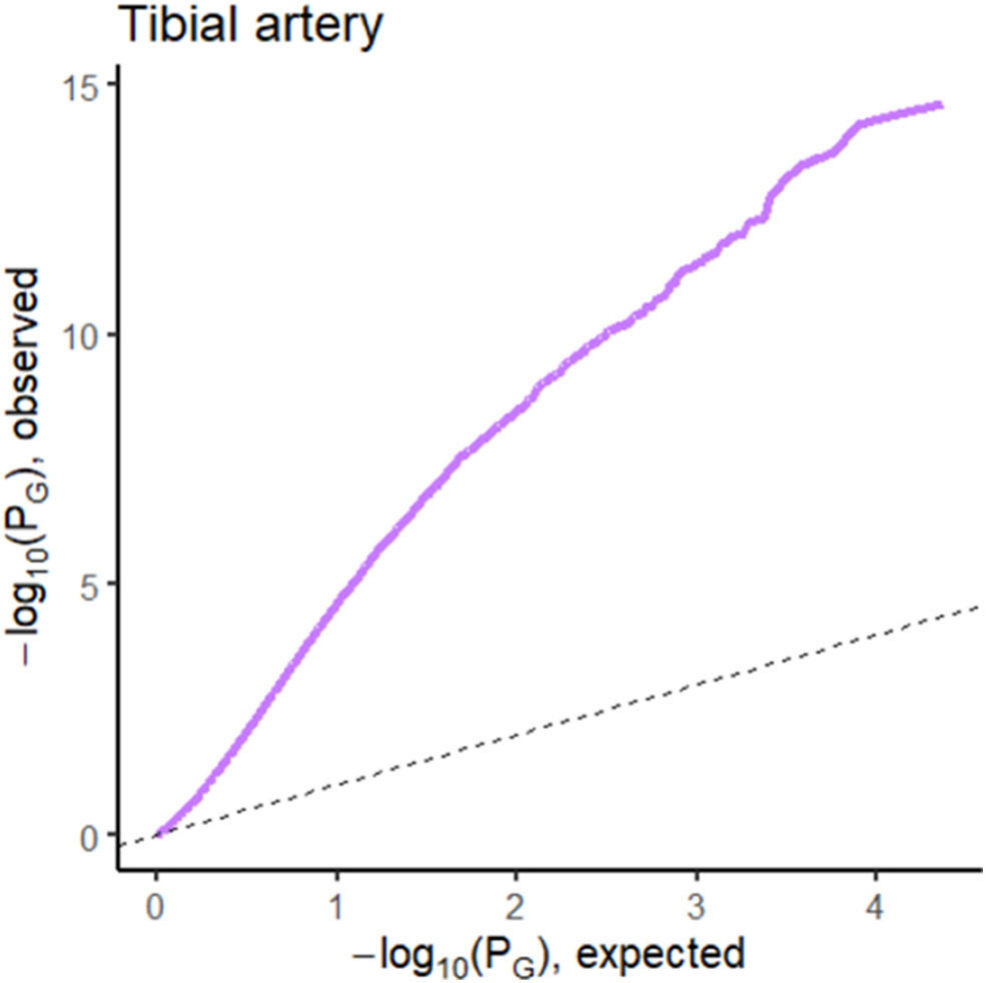
QQ plot of *p-*values from the regression analysis of pathology phenotype against gene expression for atherosclerosis in tibial artery tissue.

The prediction performance for tibial artery atherosclerosis in modest (~0.76, Table 1), again similar for imaging, expression, and combined. The imaging-based predictions are instructive. In Figure 4 (bottom), the images corresponding to the three lowest and three highest atherosclerosis probabilities are shown. The high-probability images show calcification (dark staining) portions, characteristic of the pathology (Nicoll and Henein, 2013). We note that the “error” in classification (second thyroid image from right) appears to show a classic histology pattern for the disorder. Figure 5 is an illustrative observed vs. expected qq plot of *p*-values from the regression analysis of pathology phenotype against gene expression in tibial artery, which revealed that the atherosclerosis/atherosis/sclerotic pathology is a substantial source of expression variability.

Immune pathways also topped the list of significant IPA pathways (FDR *q* < 0.1) for atherosclerosis. These included *PD-1 Cancer Immunotherapy, iCOS Signaling in* ***T****Helper Cells*, and *Allograft Rejection Signaling* (Supplementary File 1). Atherosclerosis is a chronic inflammatory disease (Galkina and Ley, 2009), with lesions containing cells involved in immune response (Hansson, 2001). Accordingly, it is perhaps not surprising that DAVID enrichment included clusters for human leukocyte antigen (HLA), interleukin (IL), and cluster of differentiation (CD). Proteins produced by HLA exist on the cell surface and the immune system uses HLAs to differentiate human cells and non-human (virus) cells (Shiina et al., 2009). Interleukins participate in the regulation of immune responses, inflammatory reactions, and formation of blood cells (Sims et al., 1988). CD molecules are also on the cell surface and provide targets for immunophenotyping of cells (Chan et al., 1988).

In Figure 3, the π_1_ value for the association of tibial artery atherosclerosis against gene expression across tissues was largest for the tibial artery.

### 4.4. Thyroid—Hashimoto's Disease

Image PCs were an excellent predictor of Hashimoto's disease, confirming the findings of Barry et al. (2018), as was gene expression. The expression-based suite AUC is a remarkable 0.96. In Figure 4 (top), images with a high prediction probability show clear lymphocytic infiltration (dark staining) portions that are characteristic of the disease (Pyzik et al., 2015).

Hashimoto's disease is understood to be an auto-immune disorder, disproportionately affecting women (Zaletel and Gaberscek, 2011). As might be expected, the most significant biological pathways for expression associated with Hashimoto's disease involved immune response, with top pathways Th1/Th2 Activation, Innate/Adaptive Immune Cell Communication, and Primary Immunodeficiency at the top of a large list of immune-related pathways with FDR *q* < 0.1 (Supplementary Files 1, 2). In addition to HLA, IL, and CD, major determinants of this classification were the immunoglobulin (IG) and toll-like receptor (TLR) gene groups. Immunoglobulins are antibodies used by the immune system to neutralize pathogens such as bacteria and viruses (Lefranc, 2014). Toll-like receptors recognize molecules derived from microbes and activate immune cell responses (Delneste et al., 2007).

In Figure 3, π_1_ for the association of Hashimoto's disease against gene expression across tissues was largest for the thyroid.

### 4.5. Adipose–Fibrosis

These images (not shown) appear to have less definition than other tissues, and the AUC increased substantially (from ~ 0.78 to 0.84) using network embedding compared to the other analyses involving expression or image alone. However, the AUC performance appears to vary somewhat across the various approaches, including the simple initial analysis and the “best of suite” analyses, and it is difficult to confidently attribute the improvement to the embedding process.

### 4.6. Overall

From Table 1, we can conclude that the AUC results are fairly similar, whether using images alone or with expression. Supplementary Table 2 provides a list of the top 20 genes in the Initial Analysis using gene expression-based prediction. Our network embedding analysis using gene expression generally resulted in slightly improved predictions (AUC) compared to the other analyses involving expression or image alone. Combined analyses using the integrated approach (imaging and expression together) failed to improve prediction accuracy, either in simple form or using the feature extraction-JIVE-random forest complex analyses. One possibility is that the prediction signal for imaging and expression were largely overlapping, and thus not able to reinforce each other or represent sufficient synergy to overcome the additional model complexity. As discussed above, Figure 3 summarizes the proportion of non-null *p*-values (π_1_ = 1 − π_0_) in the cross-tissue regression analysis of phenotype and image PCs against gene expression. The rationale is that π_1_ should reflect the degree of correspondence between each pathology and the tissue, and be relatively insensitive to issues such as sample size. Note that π_1_ for the phenotype association was generally among the largest for the same tissue corresponding to the pathology. We consider this observation to be notable, as the complexity of pathway biology and the underlying driving tissues could quite conceivably result in alternate findings. Our findings offer a complementary counterpoint to the fact that the majority of general (“non-disease”) expression QTLs are thought to be common across a variety of tissues (GTEx Consortium et al., 2020). The figure also shows the same type of plot for association of expression with imaging PCs 1–3, which sometimes roughly track the π_1_ values from phenotype association. This phenomenon is to be expected if the phenotype is directly driving much of the PC signal, or if for any reason the image morphologies are correlated with expression in a particular tissue.

## 5. Discussion

Advancements in imaging technology have worked in tandem with advances in pathology, and machine learning methods are learning to mimic or even improve upon the conclusions reached by pathologists (Bera et al., 2019). In cancer diagnostics, the availability of large sample sizes has been transformative, with a narrow purpose to improve diagnostic capability. Even with such available samples, subcategorization based on expression signatures has arguably moved faster than imaging-based analoges, although the two data sources may reinforce each other (Lundberg et al., 2017).

For traits at a subclinical level, such that biopsies would not be warranted, there have remained open questions about the capabilities of automated machine learning and the predictive ability of genomics. Previous efforts on earlier, smaller version of the GTEx data (Ash et al., 2018; Barry et al., 2018) have largely focused on the image data alone, or with limited use of expression for eQTL-related findings. Here we have used the larger and final GTEx v8, increased the number of pathologies, and provided a comprehensive treatment of both imaging and expression data in the target tissue. In addition, we have brought expression from a standard set of 30 GTEx tissues into the analysis, providing important context for cross-tissue comparisons.

In our analyses, the imaging and expression data provided little evidence that they reinforce each other in building prediction models, despite considerable effort in our model building and selection. Moreover, for the phenotypes used in limited previous publications, our imaging-based AUC is similar to the previous reports (Ash et al., 2018; Barry et al., 2018), suggesting that our conclusions are supported within the state of the art of model-building. Network embedding appears to result in some prediction improvement, but could be implemented in a “natural” form for expression data only, as our treatment of the imaging data does not readily map to network structures.

Both genomic and imaging data sources provide similar and good prediction performance when analyzed independently. We find that prediction accuracy varies widely depending on the pathology/tissue, and that, with a few exceptions, integration of the imaging and expression data offer limited improvement over either source alone. Importantly, we examined expression patterns in a comprehensive set of GTEx tissues and find that cross-tissue genomic associations tend to be lower than within-tissue. We further explored expression-based prediction limits using network embedding methods, and discuss genomic pathway discoveries in thyroid and tibial artery. Our prediction accuracy using imaging alone was similar to prior reports, for the few GTEx tissue pathologies that have previously been analyzed. Thus, in our hands, we conclude that the results support the use of genomic expression measurements for their interpretability, and the ability to generate biological hypotheses, as well as to perform direct prediction.

## Data Availability Statement

GTEx histological images are available for bulk download at the Biorepositories and Biospecimen Research Branch (BBRB) of the Cancer Diagnosis Program (CDP) (https://brd.nci.nih.gov/brd/image-search/searchhome). Pathology data for all histology samples are available on the GTEx Portal (https://www.gtexportal.org/home/histologyPage). The v8 release of RNA-Seq expression data can also be accessed on the GTEx Portal (https://www.gtexportal.org/home/datasets).

## Author Contributions

Y-HZ was the leader of this review study, wrote the manuscript, designed the data analysis, summarized the results, and software management. PG contributed to the manuscript writing, implementation of analysis, results summaries, and code summaries. ES contributed to the sections A Larger Suite of Prediction Approaches and Network Embedding Analysis. All authors contributed to the article and approved the submitted version.

## Conflict of Interest

The authors declare that the research was conducted in the absence of any commercial or financial relationships that could be construed as a potential conflict of interest.
