# Supplementary material for: Exploring the Limits of Combined Image/'omics Analysis for Non-cancer Histological Phenotypes
Source: Front Genet. 2020 Oct 23;11:555886. doi: 10.3389/fgene.2020.555886 (PMC7644963; doi:10.3389/fgene.2020.555886)
Supplement: Supplementary file 3 [file Data_Sheet_3.pdf]

Supplementary Figure S1: the area under the receiver-operator characteristic curves

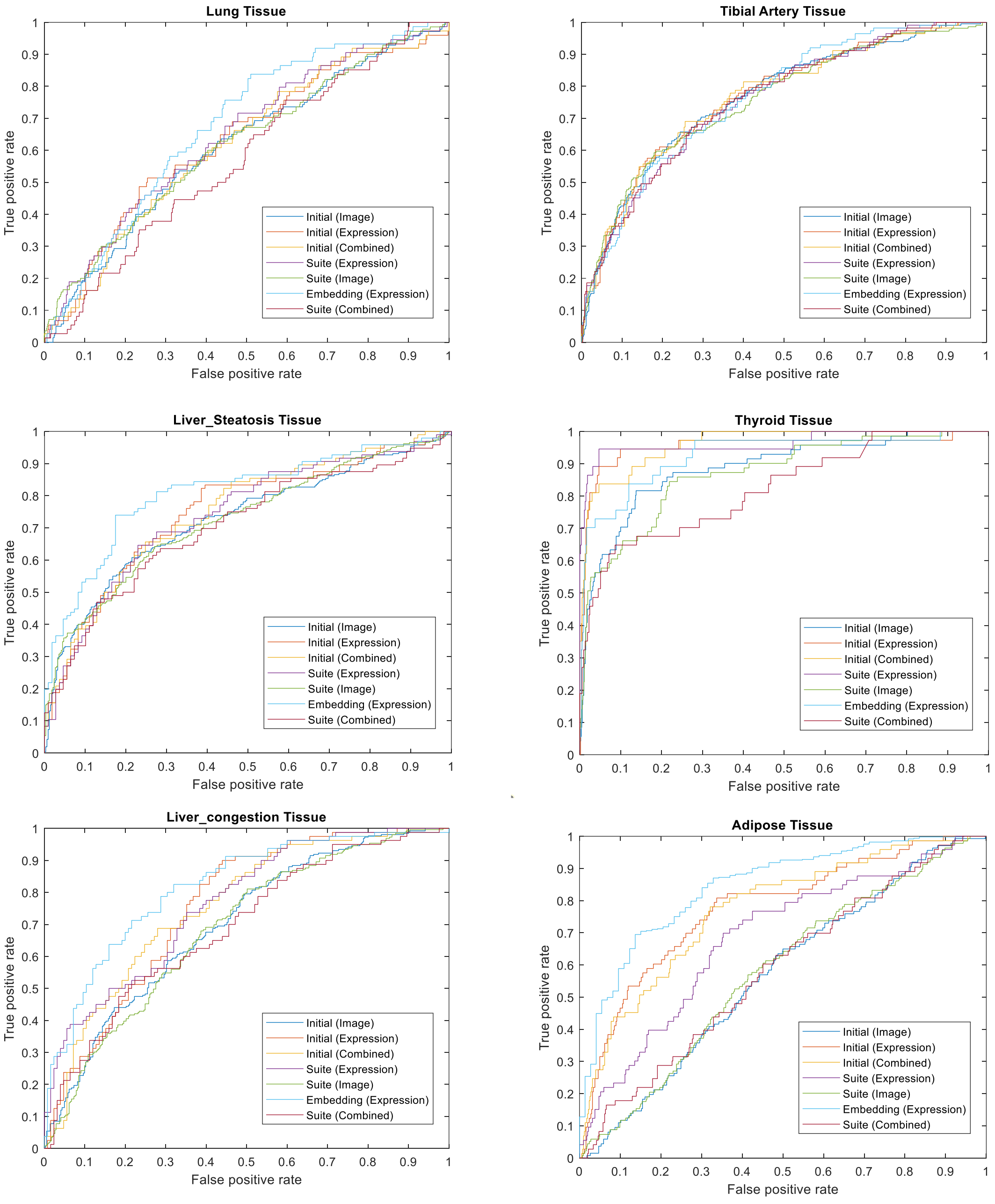

Supplementary Table 1: the size of the gene network

**Tissue-pathology**	**Gene network size** (adjacency matrix)
lung - fibrosis	4688×4688
liver - steatosis	3386 ×3386
Liver - congestion	9429 ×9429
Tibial Artery - arterosiclerosis/atherosis/sclerotic	10649×10649
Thyroid - Hashimoto	12651×12651
Adipose - fibrosis	3663×3663

Supplementary Table S2 Top 20 Genes in the initial analysis using gene expression-based prediction

	Lung - fibrosis	Liver - steatosis	Liver - congestion	Tibial artery - atherosclerosis	Thyroid - Hashimoto	Adipose - fibrosis
1	DDB2	RAP2CP1	RPSAP36	DMP1	AL928768.3	YBX1P10
2	MPP3	ARHGEF4	ARHGAP15	CDKN2A	IGLV2-18	ST13P5
3	TCF23	GRIA3	CTC-471F3.5	IBSP	PLA2G2D	MTCO3P12
4	RP11-361H10.3	REG4	HMGB3P24	GALNT3	TNFSF11	STAR
5	GRM8	SPATA18	SLC6A2	DDIT4L	PNOC	RP11-466A19.8
6	NELL1	SATB2	AC069282.6	PLEKHG3	IGLJ3	RN7SL431P
7	RP11-909N17.2	PRAMEF10	MIPEPP3	IL21R	IGLV7-43	MTATP8P1
8	RP3-333H23.9	PRAMEF33	RP1-257I20.14	XCL1	TCL1A	AC009299.5
9	SCG2	ZDHHC19	UPK1A	IVNS1ABP	IGLV3-12	RP11-867G23.1
10	CTD-2184D3.7	RP11-996F15.5	CH507-9B2.3	TEX9	CLEC17A	SLC8A3
11	DUXAP8	SPESP1	AGAP9	TNFSF11	CTC-378H22.2	RP11-513N24.1
12	BSN-AS2	CACHD1	SDK1	CHST11	IGKV6-21	ABT1
13	TRBV13	TSSK5P	LRRC36	DGKA	IGKV3D-15	CTD-2366F13.2
14	CDC37P1	MGARP	ZNF300P1	SNX21	FAM46C	AC069513.4
15	CXorf67	RP11-767C1.2	HRC	FRMD4B	TNFRSF13B	ANKRD2
16	RP11-715J22.1	BEX1	TRIM26	SLCO3A1	IGLC7	GLOD4
17	ST13P20	RP11-157J24.2	PRELID1P4	FAM234A	IGKV1D-12	CTA-363E19.2
18	CCT6P3	THRAP3	IL10	LINC01535	MIXL1	AC004951.6
19	GNG4	CFAP99	PDE5A	DLX2	TRBV4-2	CTD-2647L4.4
20	HECW1	RASL10B	KIAA1468	ARHGAP5-AS1	IGKV3-7	SNORA65
